# Diagnostic anténatal d’un rachischisis

**DOI:** 10.11604/pamj.2016.25.191.10912

**Published:** 2016-11-25

**Authors:** Amira Ayachi, Mechaal Mourali

**Affiliations:** 1 Service de Gynécologie et Obstétrique, Unité De Diagnostic Anténatal, Faculté de Médecine de Tunis, Université Tunis El Manar, CHU Bougatfa, Bizerte, Tunisie

**Keywords:** Diagnostic anténatal, rachischisis, spina bifida, Prenatal diagnosis, rachischisis, spina bifida

## Image en médecine

Il s’agit d’une patiente âgée de 30 ans, G1P0, adressée à notre unité de diagnostic anténatal pour suspicion de spina bifida aperta à un terme de 24 SA+2jr. L’échographie du premier trimestre réévaluée ne répondait aux critères qualités permettant de faire un diagnostic précoce. Les marqueurs sériques du premier trimestre prescrits, n’ont pas été réalisés par la patiente. L’échographie morphologique retrouve un diamètre bipariétal inférieur au 3^ème^ percentile avec une dépression des os temporaux (A), une absence de visualisation de la grande citerne, un Chiari II avec un cervelet plongeant dans la fosse postérieure B) et une anomalie de fermeture du tube neural à type de rachischisis (C). Une échographie en mode tridimensionnelle a été réalisée visualisant le défect dorsolombaire étendu (D), mais persistance de mouvements des membres inférieurs jugés satisfaisants. Après informations délivrées au couple sur le pronostic sombre des anomalies échographiques constatées, le couple a décidé d’interrompre la grossesse E) après réalisation d’un caryotype sur liquide amniotique. La formule chromosomique était normale: 46, XY. A la sortie, une ordonnance de supplémentation en acide folique a été délivrée à la patiente.

**Figure 1:**
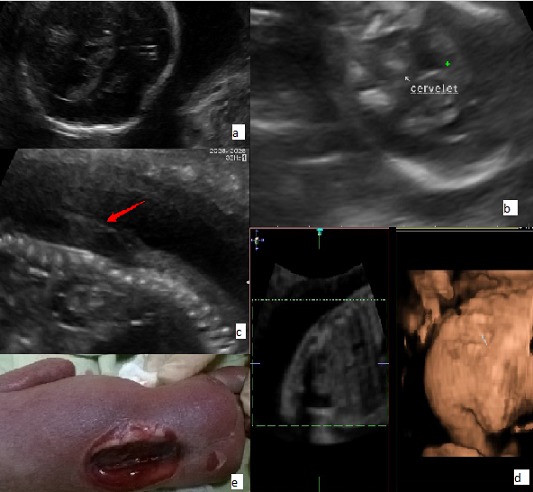
(A) diamètre bipariétal inférieur au 3^ème^ percentile avec une dépression des os temporaux; (B) cervelet plongeant dans la fosse postérieure; (C) rachischisis; (D) rachischisis en mode 3D; (E) rachischisis après IMG

